# Synthesis of Lysophospholipids

**DOI:** 10.3390/molecules15031354

**Published:** 2010-03-08

**Authors:** Paola D’Arrigo, Stefano Servi

**Affiliations:** 1 Dipartimento di Chimica, Materiali ed Ingegneria Chimica “Giulio Natta”, Politecnico di Milano, Via Mancinelli 7, 20131 Milano, Italy; 2 Centro Interuniversitario di Ricerca in Biotecnologie Proteiche "The Protein Factory", Politecnico di Milano and Università degli Studi dell'Insubria, Via Mancinelli 7, 20131 Milano, Italy

**Keywords:** lysophospholipids, biocatalysis, phospholipase, chemo-enzymatic synthesis, lysophosphatidylcholine, lysophosphatidic acid

## Abstract

New synthetic methods for the preparation of biologically active phospholipids and lysophospholipids (LPLs) are very important in solving problems of membrane–chemistry and biochemistry. Traditionally considered just as second-messenger molecules regulating intracellular signalling pathways, LPLs have recently shown to be involved in many physiological and pathological processes such as inflammation, reproduction, angiogenesis, tumorogenesis, atherosclerosis and nervous system regulation. Elucidation of the mechanistic details involved in the enzymological, cell-biological and membrane-biophysical roles of LPLs relies obviously on the availability of structurally diverse compounds. A variety of chemical and enzymatic routes have been reported in the literature for the synthesis of LPLs: the enzymatic transformation of natural glycerophospholipids (GPLs) using regiospecific enzymes such as phospholipases A_1_ (PLA_1_), A_2_ (PLA_2_) phospholipase D (PLD) and different lipases, the coupling of enzymatic processes with chemical transformations, the complete chemical synthesis of LPLs starting from glycerol or derivatives. In this review, chemo-enzymatic procedures leading to 1- and 2-LPLs will be described.

## Abbreviations

PL: phospholipid
LPL: lysophospholipid
GPC: *sn*-glycero-3-phosphorylcholine
LPA: lysophosphatidic acid
LPC: lysophosphatidylcholine

## 1. Introduction

Development of new synthetic methods for the preparation of biologically active phospholipid derivatives is a challenging problem of membrane-chemistry and biochemistry today. In fact structural and dynamic studies of biomembranes for the establishment of structure-activity relationships, phospholipids-proteins interactions and mechanisms of action of phospholipids metabolizing enzymes require the preparation of a great number of phospholipids derivatives as the key step in advancing membrane biochemistry.

Lysophospholipids have recently become the focus of special attention since it was discovered that in addition to their role in phospholipid metabolism they function as second messengers, exhibiting a broad range of biological activities in their own right. Moreover phospholipids analogues and lysophospholipids incorporating spectroscopically active groups have been shown to be structural probes to study the aggregation properties of these particular compounds and the fate of products generated by phospholipids metabolizing enzymes.

### 1.1. Phospholipids (PLs)

Phospholipids (PLs) are critical constituents of most biological membranes; they are amphipathic in nature and their aggregation properties are the focus of intense study [1,2,3]. PLs are characterized by a glycerol backbone to which a polar phosphodiester group is linked at the *sn*-3 carbon, while the polar head group defines the class of the PLs. Further, two fatty acid-derived acyl residues are linked at the *sn*-1 and *sn*-2 carbons. The structural diversity of PLs is attributable to different polar head group, fatty acids substituents and their regioisomerism. They can be thought of as having three distinct structural regions: (a) a polar hydrophilic headgroup which resides at the lipid-water interface when assembled into organized structures; (b) an interfacial region which is of intermediate polarity; (c) a hydrophobic tail region.

PLs, essential constituents of cellular membranes, have been thoroughly investigated and are the topic of many areas of biomedical research [4,5,6]. Recent advances in the construction of artificial cell membranes with specific biological functions require tailor-made glycerol-derived lipids [7]. In addition to their natural biological role, PLs are used for several practical applications, such as emulsification in pharmaceuticals and food and preparation of liposomes for cosmetics and drug delivery [8,9].

### 1.2. Lysophospholipids (LPLs)

**Figure 1:**
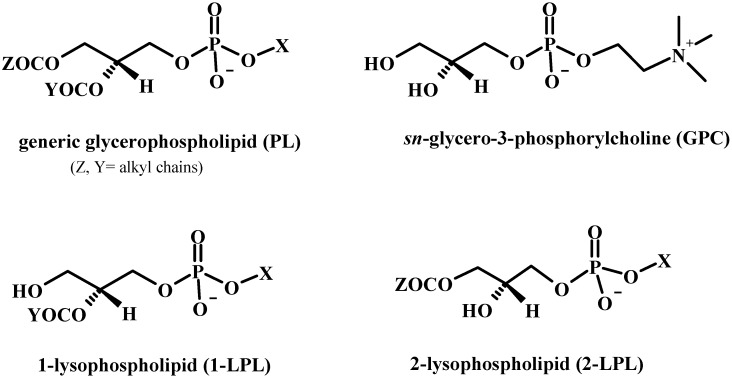
Structures of phospholipids and derivatives.

Lysophospholipids (LPLs) are glycerophospholipids in which one acyl chain is lacking and then only one hydroxyl group of the glycerol backbone is acylated (see Figure 1). 1-Lysophospholipids (1-LPLs) maintain the acyl chain in position 2, whereas 2-lysophospholipids (2-LPLs) are only acylated at position 1. LPLs possess properties similar to PLs: they are good emulsifying and solubilising agents and useful synthetic intermediates for the preparation of PLs for applications in foods, cosmetics, agrochemicals and pharmaceuticals [10,11]. Unlike PLs, LPLs are found only in small amounts in biological cell membranes [12]. LPLs are membrane-derived signalling molecules produced by phospholipases that exhibit a wide range of diverse biological activities [13,14]. In fact LPLs and their receptors have been found in a wide range of tissues and cell types, indicating their importance in many physiological processes including reproduction [15], vascular development [16] and nervous system [17]. They play different critical and incompletely elucidated roles in development and diseases [18]. Over the past decade, it has become clear that medically relevant LPLs activities are mediated by specific G protein-coupled receptor (GPCR), implicating them in the etiology of a growing number of disorders, such as inflammation, autoimmune diseases, neuropathic pain, atherosclerosis, cancer and obesity [19,20,21,22,23,24,25,26,27,28,29,30,31]. 

### 1.3. 2-Lysophosphatidylcholine (2-LPC)

2-Lysophosphatidylcholine (2-LPC) is the best-studied LPL, being the most abundant LPL in Nature. It has been shown to be involved in the regulation of gene transcription, mitogenesis, monocyte chemotaxis, smooth muscle relaxation and platelet activation [25,32]. Many reviews describe its regulatory effects [33]. For example 2-LPC is directly involved in signal transduction pathway through protein kinase C to induce long-term cellular responses, it accumulates in tissues during ischemia and in plasma of inflammatory arthritis [34]. When released from the liver as a product of phospholipase A_2_ hydrolysis or produced by the enzyme lecithin/cholesterol acyl transferase in the plasma, 2-LPC is converted by lysophospholipase D (Lyso-PLD) to lysophosphatidic acid (2-LPA, 1-*O*-acyl-*sn*-glycero-3-phosphate), a highly potent inducer of cell proliferation, migration and survival [35,36].

**Figure 2:**
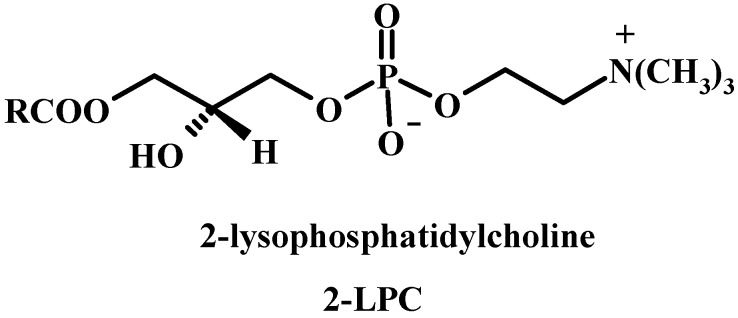
Structure of 2-lysophosphatidylcholine.

### 1.4. Lysophosphatidic acid (LPA)

LPA has been shown to act as an important intermediate in transmembrane signal transduction processes as a platelet activating factor and in the stimulation of cell proliferation [37]. The recent identification and cloning of GPCRs having high affinity for LPA and another important LPL sphingosine 1-phosphate (S1P) have enabled a greater mechanistic understanding of their diverse roles in biological processes [38,39]. LPA and S1P regulate the development and function of numerous organ systems, including the cardiovascular [40], nervous [41], immune [42] and reproductive systems [43].

**Figure 3:**
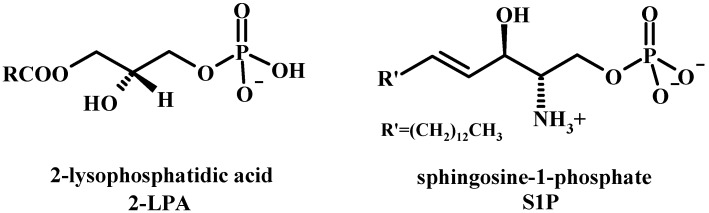
Structure of 2-lysophosphatidic acid and sphingosine-1-phosphate.

### 1.5. LPLs availability

The ability to obtain reliable data on the physiological activities of LPLs is hampered by the lack of availability and their metabolic lability. Elucidation of the mechanistic details involved in enzymological, cell-biological and membrane-biophysical activities of LPLs remains to be accomplished and it greatly depends on availability of synthetic methods for the preparation of structurally variable LPL derivatives. Along these lines, spectroscopically labelled LPCs should become useful mechanistic probes for studying the enzymes involved in LPL biosynthesis and degradation, understanding LPLs self-assembly, including their interaction with membrane bilayers and monitoring the fate of the compounds in cell cultures and tissues. Also LPLs bearing fluorescent groups could offer an attractive alternative to radioactive compounds. 

To date relatively few synthetic methods have been developed for the preparation of LPLs, mainly due to the difficulties associated with a multi-step process starting from enantiomerically pure glycerol derivatives through tedious protection-deprotection steps and the possible acyl- and phosphoryl-group migration during the course of the syntheses, leading to regioisomeric mixtures of products.

## 2. Chemical Synthesis

The major challenge in LPL synthesis is the construction of the chiral structure and the conservation of the configuration in the further chemical processing. That’s why the chemical synthesis of optically active LPLs involves the extensive use of protecting groups and requires considerable expertise in synthetic lipid chemistry. Intramolecular acyl group migration in fact results in a mixture of 1-acyl and 2-acyl-glycerophosphates derivatives. Acyl migration is a problem often encountered in selective synthesis of LPL regioisomers. It consists of the intramolecular transfer of one fatty acid moiety from one hydroxyl group to the adjacent one. It is a non-enzymatic reaction which can be catalyzed by acids and bases. It is possible to slow or to suppress this acyl migration with the control of the pH or with some special reagents such as the addition of borate [44]. 2-LPC, the regioisomer deprived of the acyl chain in position 2 is much more stable than the 1-LPC isomer; the equilibrium at 25 °C between the two compounds being approximatively 9:1 [45]. 

**Figure 4:**
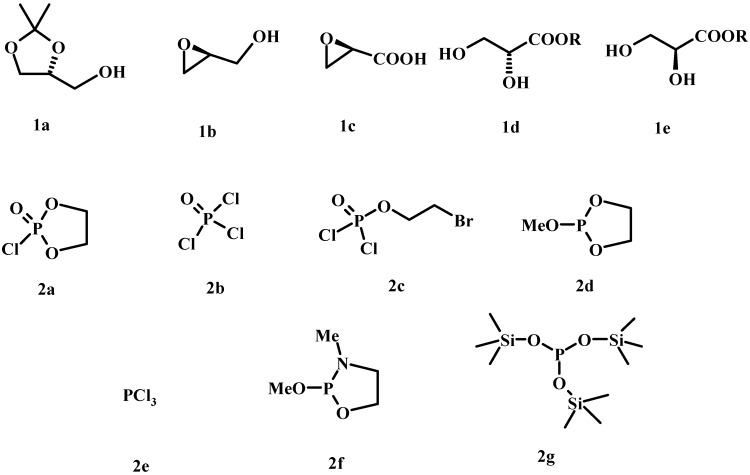
C-3 Building blocks (1a-e) and phosphorylating agents (2a-2g) most commonly used for the assembly of LPLs.

Since LPLs can be obtained from the corresponding PLs by selective enzymatic hydrolysis of one acyl chain, the total synthesis of PLs can also give access to LPLs and have been reported [46]. As in the synthesis of PLs, optically pure glycerol derivatives as C-3 building blocks are powerful starting materials also for the synthesis of LPLs. The general procedure includes then the preparation of stereospecific acyl or ether-substituted glycerol backbone and further phosphorylation of glycerol derivatives. Figure 4 shows the building blocks most commonly used for the assembly of LPLs.

### 2.1. From D-mannitol

**Figure 5:**
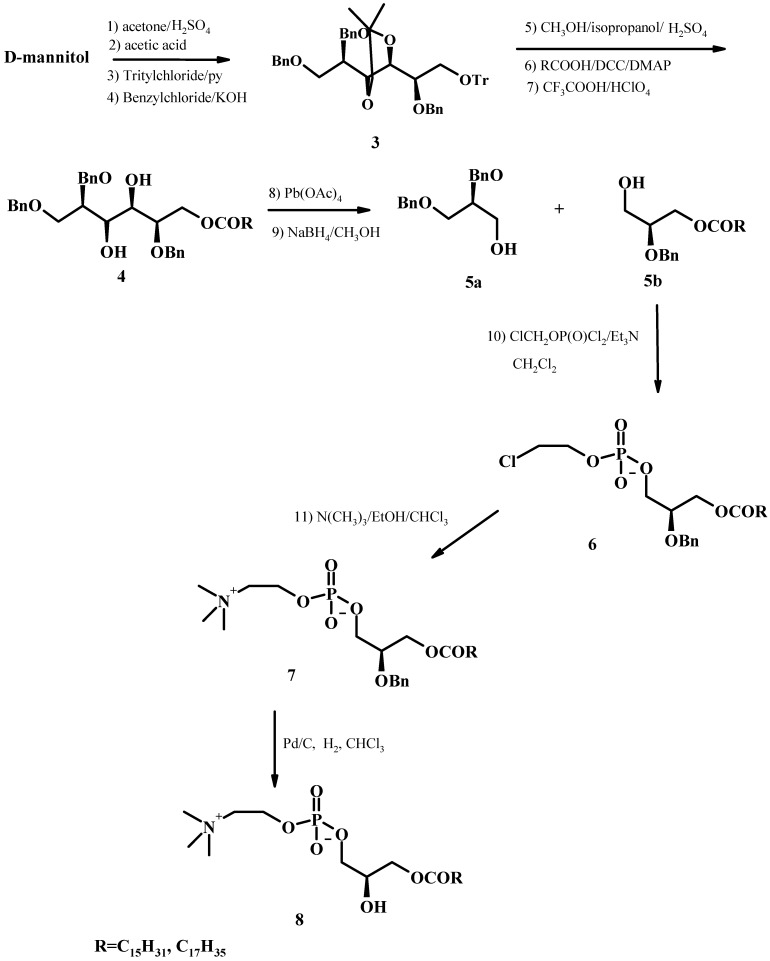
Synthesis of 2-LPC from D-mannitol.

D-Mannitol is a convenient optically active material which can be transformed through several reaction steps into LPLs [47]. D-mannitol is firstly transformed in 1,2,3,4,5,6-triisopropylidene-D-mannitol with acetone/H_2_SO_4_, then ditritylated with dry trityl chloride in anhydrous pyridine. The main product, the monotrityl derivative, is then benzylated to give compound **3**. The trityl group is selectively removed from compound **3** in methanol/2-propanol/H_2_SO_4_ to obtain 2,5,6-tribenzyl-3,4-isopropylidene-D-mannitol, which was then esterified in dry CH_2_Cl_2_ with saturated fatty acid and dicyclohexylcarbodiimide (DCC) and catalytic amount of 4-dimethylaminopyridine (DMAP) to produce 2,5,6-tribenzyl-1-acyl-3,4-isopropylidene-D-mannitol. After treatment with CH_2_Cl_2_-TFA 70% HClO_4_, product **4** is treated with lead tetraacetate in dry ethyl acetate and the resulting aldehyde then reduced with NaBH_4 _to give products **5a** and **5b**. Product **5b** is phosphorylated with cholorethylphosphoric acid dichloride in dry CH_2_Cl_2_ and anhydrous triethylamine yielding product **6** with natural configuration. The final product is aminated with trimethylamine in CHCl_3_-Ethanol and dibenzylated in presence of Pd-C(10%) and H_2_ to give the final LPC **8** (see Figure 5).

In this synthesis compound **5b** is obtained from mannitol through several chemical steps. Three of the six carbon atoms of the starting material are sacrified and the synthon contains only one of the four chiral centers of the sugar. Transformation into the LPL **8** requires phosphorylation with cholorethylphosphoric acid dichloride, one of the synthons used in phosphorylation and two more reaction steps. This synthesis is rather typical of synthetic methods requiring many steps and affording low yields.

### 2.2. From glycidol

**Figure 6:**
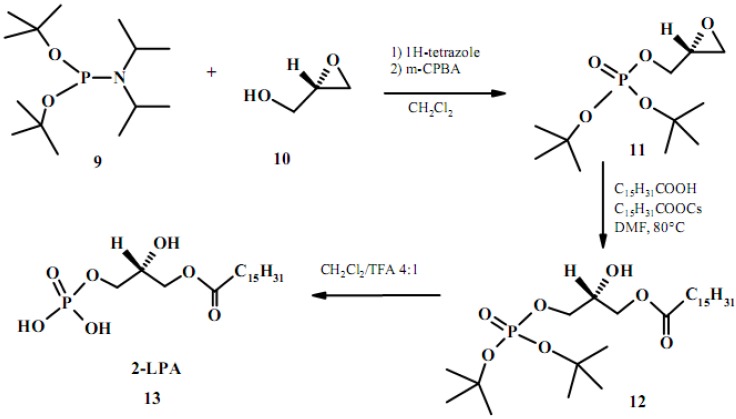
Efficient synthesis of 2-LPA starting from (*S*)-glycidol (**10**).

**Figure 7:**
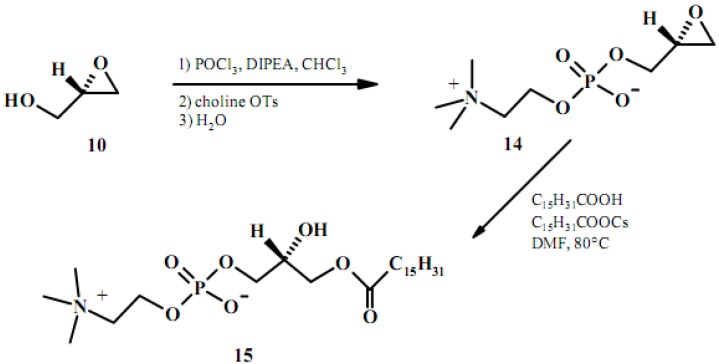
Synthesis of 2-LPC from (*S*)-glycidol.

Enantiomerically pure glycidol is an alternative to chiral glycerol derivatives and is in general obtained from the same starting materials (carbohydrates). One of the synthesis starting from this C-3 synthon relies on nucleophilic ring opening of phosphorylated glycidol derivatives, yielding only minor amounts of the migrated *sn*-2-acyl by-product and providing a rather efficient sequence to LPLs [48]. In this procedure phosphorylation of commercially available (*S*)-glycidol (**10**) was performed with di-*tert*-butyl-*N,N*-diisopropyl phosphoroamidite (**9**) and subsequent oxidation using *m*-CPBA to produce the derivative (*R*)-di-*tert-*butyl*-*phosphorylglycidol (**11**) in 74% yield (see Figure 6). Regioselective opening of the epoxide with cesium palmitate produced **12** in 71% yield. Treatment of **12** with TFA in CH_2_Cl_2_ cleanly produced the deprotected 2-LPA **13** in quantitative yield. The overall yield was 46%. During the reaction sequence minor amounts of the regioisomeric 2-*O*-palmitoyl derivative was produced by acyl migration from the primary position.

(*S*)-Glycidol (**10**) can alternatively be phosphorylated using phosphorous oxychloride and subsequently treated with choline tosylate and pyridine to produce the choline derivative **14** in 53% yield. The mucleophilic opening of the epoxide with cesium palmitate produce 2- LPC **15** (Figure 7). 

The enantiomeric forms of LPA and LPC can analogously be obtained from (*R*)-glycidol. Thus with this method both natural and unnatural enantiomers of a broad range of biologically important LPLs can be obtained in high optical purity and yields. 

### 2.3. From glyceric acid

**Figure 8:**
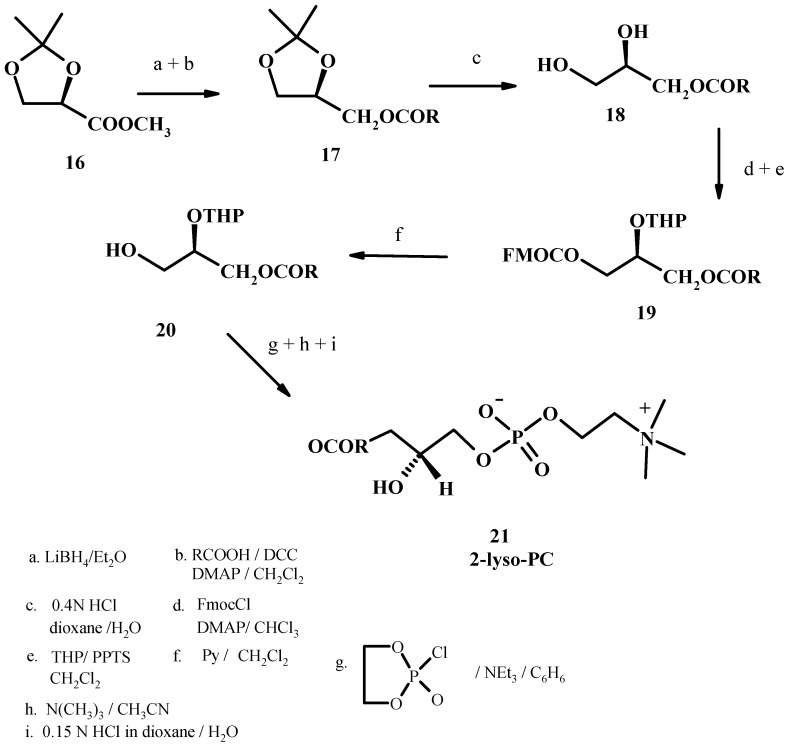
A stereospecific synthesis of LPC starting from glyceric acid.

This method consists in a stereospecific synthesis of LPCs based on orthogonal protection of hydroxyl group derived from glyceric acid using fluorenylmethylcarbonate [49]. Briefly the reduction of 2,3-*O*-isopropylidene-L-methylglycerate (**16**) with LiBH_4_ in ether yielded the corresponding alcohol which was acylated with palmitic acid/DCC and DMAP. After purification of **17**, acid-catalyzed deprotection and chromatography gives 1-palmitoyl-*sn*-glycerol (**18**). Regioselective monoacylation of **18** was accomplished using FMOC-chloroformate and DMAP. Tetrahydropyranylation of the *sn-*2 hydroxyl group and subsequent cleavage of the FMOC-carbonate function give compound **20** with 87% yield. After phosphorylation and removal of the protecting group, the desired 1-palmitoyl-*sn*-glycerophosphocholine (**21**) was obtained (see Figure 8).

### 2.4. Use of p-nitrophenyl glycerate

**Figure 9:**
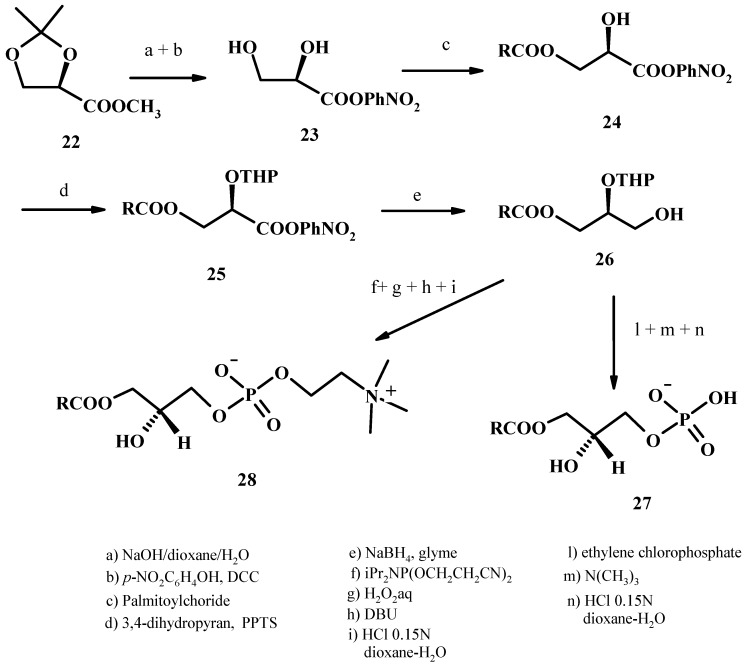
Synthesis of 2-LPC from *p*-nitrophenylglycerate.

This synthesis starts from the *p*-nitrophenylester of D-glyceric acid which is obtained from the commercially available 2,3-*O*-isopropylidene-D-glyceric acid methyl ester (**22**) by reaction with NaOH in aqueous dioxane, followed by DCC promoted condensation with *p*-nitrophenol and subsequent acidolytic cleavage of the isopropylidene function to give the active ester of the D-glyceric acid **23** in an overall yield of 66% [50]. Regiospecific monocylation of compound **23** at the primary alcohol function was accomplished using two-fold molar excess of palmitoyl chloride in a mixture of CH_3_CN/benzene (1/1) at room temperature for 72h. The pure *sn*-1 palmitoyl ester **24** was obtained after chromatography on Sephadex LH-20 column. Tetrahydropyranylation at the *sn*-2 glycerol position was carried out with 3,4-dihydropyran in CHCl_3_ in presence of pyridinium *p*-toluensolfonate (PPTS) as catalyst at r.t. Product **25** was then reduced with excess of NaBH_4_ in 1,2-dimethoxyethane at r.t. to give 1-palmitoyl-2-tetrahydropyranyl-*sn*-glycerol (**26**). With readily available phosphorylation methods and cleavage of the *sn*-2 tetrahydropyranyl group the glycerol derivative **26** has been easily transformed in LPA **27** and LPC **28** (see Figure 9). Under these conditions no acyl migration takes place.

### 2.5. Use of p-toluensulfonate for hydroxyl group protection

This new stereoselective synthesis of LPCs is based on: (1) the use of 3-*p*-toluenesulfonyl-*sn*-glycerol (**29**) (prepared from acid-catalyzed deprotection of commercially available 1,2-*O*-isopropylidene-*sn*-glyceryl-3-tosylate using 0.4N HCl in methanol) to provide the stereocenter for construction of the optically active LPL molecule **33**; (2) tetrahydropyranylation of the secondary alcohol function to achieve orthogonal protection of the *sn*-2 and *sn*-3 glycerol positions (product **31**), and (3) elaboration of the phosphodiester headgroup using a cyclic phosphochloridate, the 2-chloro-1,3,2-dioxaphospholane, and trimethylamine [51]. It has also been found that the conversion of the tosyl group into the hydroxyl in compound **32** can be achieved via metoxyacetate displacement of the *sn*-3-p-toluenesulphonate cleaving the reactive methoxyacetyl ester with methanol/*tert*-butylamine. In these conditions the ester group at the *sn*-1 position remains unaffected. Because of the order in which the functional groups are introduced this synthetic strategy requires minimal use of protecting groups. The sequence has been shown to be suitable for the preparation of spectroscopically labelled LPCs.

**Figure 10:**
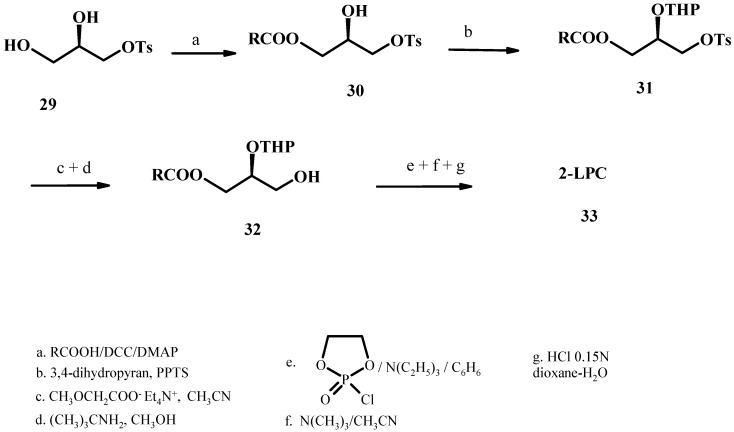
Synthesis of 2-LPC starting from 3-*p*-toluenesulfonyl-*sn*-glycerol.

### 2.6. From sn-glycero-3-phosphorylcholine

*sn*-Glycero-3-phosphorylcholine (GPC), readily available from alcoholysis of natural lecithins [52], is the ideal starting material for the preparation of PLs of various type since it has the same chirality that PLs have in nature. However GPC is soluble just in water or in lower alcohols such as methanol whereas it presents a low solubility in organic solvents: this characteristic has hampered its use in synthesis. Obviously, alcohols can not be used as solvents for esterification of the hydroxyl group of GPC. Most of the methods for the GPC acylation employ the cadmium chloride complex of GPC which is much more soluble in DMF or other polar organic solvents [53]. However the use of a heavy metal such as cadmium appears not suitable for the larger scale synthesis of derivatives because heavy metals can be carcinogenic, persist in the environment and their bioaccumulation takes place in the food chain [54]. We have recently discovered that mono- and regio-selective acylation of GPC can be readily achieved by tin technology [55]. In fact the monoacylation at position 1 of GPC can be performed by a tin-mediated acylation, exploiting the high reactivity and selectivity of stannylene ketals formed *in situ*. 

**Figure 11:**
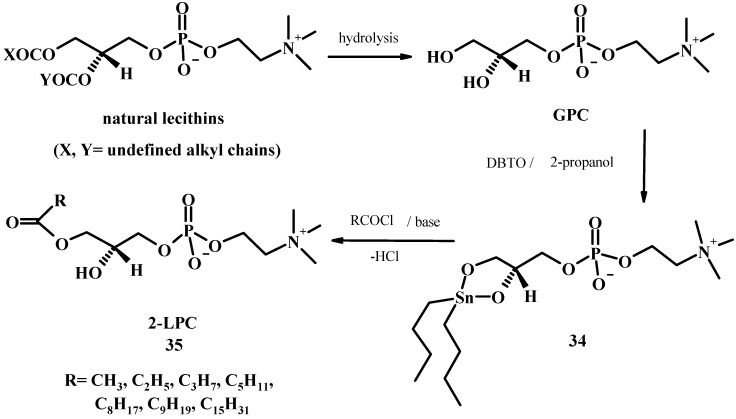
Tin-mediated synthesis of 2-LPC from natural lecithins.

The method consists of the formation of a stannylene derivative **34** in 2-propanol and subsequent acylation with a stoichiometric amount of acylating agent, resulting in the exclusive formation of 1-acyl-2-lysophospholipid **35** (see Figure 11). The advantages of the tin-mediated monofunctionalisation of the GPC are the high regio- and chemoselectivity and the short time reaction compared with conventional chemical acylation. When compared to others methods of mono-functionalisation of GPCs, this approach gives mono-acyl derivatives free from the regioisomer usually produced by reaction protocols involving the use of acidic conditions during the reaction or in the isolation procedures. The possibility of forming the stannylene ketal and the successive acylation reaction in a protic solvent that does not interfere with the acylation reaction is the key point of the procedure. The method is general, giving comparable yields with saturated and unsaturated fatty acid derivatives of different lengths [56]. The method is applicable also to the acylation of glycerol-3-phosphate (GPA). Glycerol-3-phosphate disodium salt, insoluble in 2-propanol, was firstly transformed into the mono-DMAP salt to improve its solubility. Complete removal of the metal catalyst is easily achieved by extraction procedures.

## 3. Enzymatic Synthesis

Comparing to chemical methods, enzymatic synthesis of LPLs has many advantages. In fact there are several good reasons why a biocatalytic approach to structurally defined LPLs is desirable: the first concerns the selectivity or specificity of enzymes, the second the reduction of the amount of chemical reagents, often toxic and deleterious, to be used in the synthetic steps and the mild reaction condition. The third reason is the easier purification procedure due to the intrinsic selectivity and specificity of the enzymatic catalysis. In fact fewer by-products will be formed. Another consideration associated with LPLs purification is that in case of incomplete reaction, possible impurities in the final product will constitute a much more acceptable problem if starting materials are compounds already GRAS (generally regarded as safe) as natural PLs and enzymes are, than in the case of residual chemical reagents. The potential of PL modifying enzymes appears therefore of great interest in synthesis, particularly on a industrial scale. 

**Figure 12:**
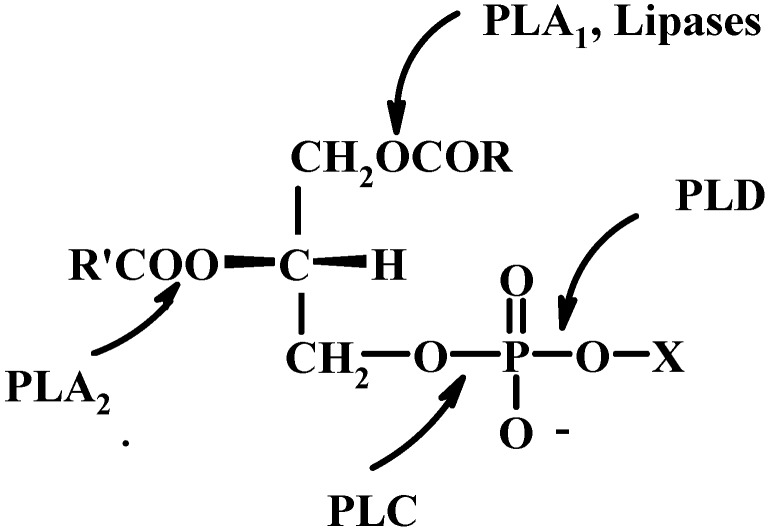
Enzymes acting on phospholipids and their specific site of action.

Natural PLs are the natural precursors of LPLs. They have two carboxylic esters bonds and two phosphates ester bonds. Therefore selective recognition requires regio- and chemoselective properties. The enzymes which selectively catalyze the transformation of PLs are named phospholipases [57,58]. Phospholipases play crucial roles in cellular regulation, metabolism, and biosynthesis of PLs. Each of the four major phospholipases selectively recognize one of the four ester bonds (see Figure 12). Phospholipases are ubiquitous enzymes occurring from mammals to bacteria. At present, nearly hundred phospholipases have been purified, characterized and cloned [59,60,61]. Beside phospholipases, there are many other enzymes that can be used for the modification of PLs. One of the big groups of such enzymes is constituted by lipases, non-specific enzymes with a broad substrate specificity. Lipases have been more deeply developed and studied than phospholipases in terms of both theoretical and practical understanding.

Detailed studies of the structure, mechanism and kinetics of phospholipases have been reviewed [62]. In biocatalysis, phospholipases have been exploited for a number of PL transforming reactions in the laboratory as well as on an industrial scale [63,64,65,66]. It should also be pointed out that enzymatic catalysis provides routes for obtaining PLs and LPLs with the natural configuration at the *sn-*2 centre. 

As already reported in the introduction, because of their superior emulsification properties, LPLs have numerous applications in the food, cosmetic, pharmaceuticals industries. However their properties depend strongly on the fatty acid component and on the polar head bound to the glycerol backbone. By changing the hydrophilic/lipophilic balance in these compounds using lipases or phospholipases, it is possible to produce tailor-made derivatives for specific applications.

We will discuss the use of these enzymes for the synthesis of LPLs excluding the phospholipase C (PLC, phosphatidylcholine cholinephosphohydrolase, E.C. 3.1.4.3) which is specific of the hydrolysis of the phosphate ester linked to the glycerol backbone and then not useful for our aims (see Figure 12). 

### 3.1. Use of phospholipases A

Type A phospholipases are named according to their positional specificity on PL molecule, the A_1_ type catalyzing the hydrolytic removal of the acyl moiety at the *sn*-1 position (giving 1-LPL), the A_2_ type removing the acyl group at the *sn*-2 position of the glyceride molecule (giving 2-LPL). Both enzymatic activities have been identified in a large variety of cells and tissues and are probably ubiquitous. 

#### 3.1.1. Phospholipase A_1_ (PLA_1_) and lipases

Phospholipase A_1_ (PLA_1_, phosphatidylcholine 1-acylhydrolase, EC 3.1.1.32) hydrolyzes PLs at the *sn*-1 position giving a fatty acid and a 1-LPL as products; however the few commercially available enzymes of this type have not been used extensively in PLs modifications [67]. The same activity is also present in a number of ester hydrolases (lipases triacylglycerol acyl hydrolase E.C. 3.1.1.3) of different origin such as guinea pig pancreatic lipases, *Rhizopus arrhizus*, in *Aspergillus oryzae*, in *Rhizomucor miehei, Mucor javanicus*, *A. niger* and others [68,69,70,71,72,73,74,75,76]. As these enzymes are much more accessible than PLA_1_ they provide a useful alternative. 

#### 3.1.2. Phospholipase A_2_ (PLA_2_)

Phospholipase A_2_ (PLA_2_, phosphatidylcholine 2-acylhydrolase, EC 3.1.1.4) hydrolyses phosphatidylcholine PC to 2-LPC and fatty acid. The enzyme often used for biocatalytic applications is found in invertebrates (bee), in the venom of snakes (*Crotalus adamanteus*, *Naja naja*, *Crotalus atrox*), in mammals (bovine pancreas, porcine pancreas) [77] and in microorganisms (*Streptomyces violaceoruber, S. cinnamomeus, S. griseus)* [78,79]*.* The phospholipases A_2_ in venoms of several animal species as well as the digestive enzyme produced by the pancreas are by far the most thoroughly investigated enzymes of this type.

Use of this enzyme allows the removal/replacement of the acyl chain at the position *sn-*2 either via hydrolysis and subsequent chemical reesterification or through direct transesterification with an acyl donor. Hydrolysis reactions are usually more efficient than transesterifications. Quantitative removal of the acyl chain in position *sn-*2 can be performed in mixed micelles but biphasic systems are preferred because of the easier isolation of the product [80].

#### 3.1.3. Enzymatic production of LPLs using PLA_1_, PLA_2_ and lipases

**Figure 13:**
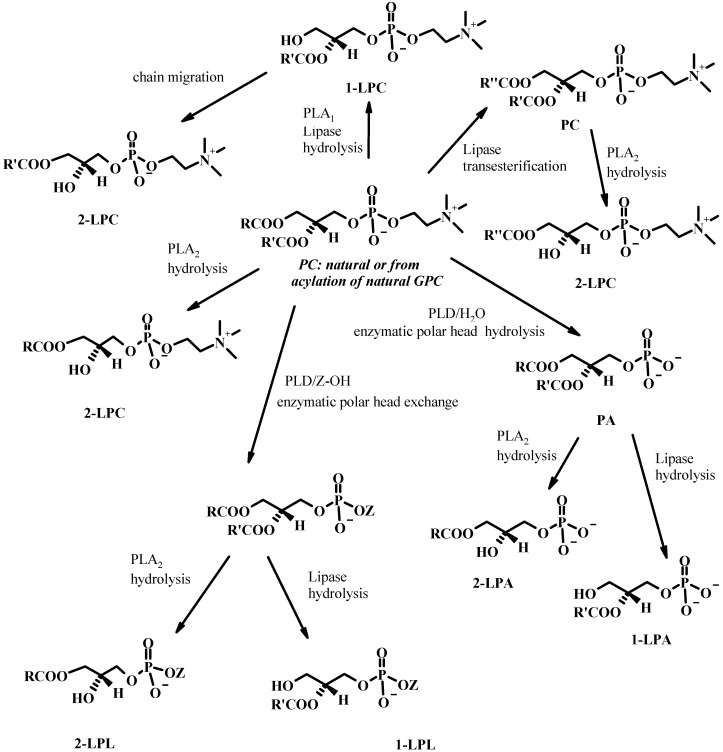
Phospholipases/Lipases catalyzed transformations of phospholipids.

All these enzymes can be used in hydrolysis reaction of PLs or in reactions of alcoholysis of PLs, or esterification of GPC (see Figure 13). 2-LPA and 2-LPC can be obtained easily from natural PA or PC by hydrolysis with PLA_2_. The enzymatic alcoholysis of PLs in the presence of lipases can produce simultaneously 1-LPLs and fatty acid esters. The starting alcohol can be glycerol, methanol or ethanol for example [81]. The direction of the reaction depends on the equilibrium costant and the reactant concentrations [66].

Another method is the enzymatic synthesis of LPC by esterification of GPC with fatty acid anhydrides or with free fatty acid in a solvent free system [82,83]. 2-LPL can be obtained in the presence of one of the lipases that shows the appropriate selectivity. Also different works focus their attention on LPC synthesis with immobilized lipase B from *Candida antarctica* (Novozym 435) starting from GPC and different vinyl esters of palmitic, capric, caprylic and lauric acids [84,28]: the lipase was selective for the *sn-*1 position of the glycerol backbone, and almost no PC was produced in the first 24 hours of the reaction. The reaction temperature has to be controlled to improve conversion and purity of the formed products. A high excess of fatty acid vinyl ester to GPC (>10 times) is also necessary to achieve significant conversion. 

**Figure 14:**
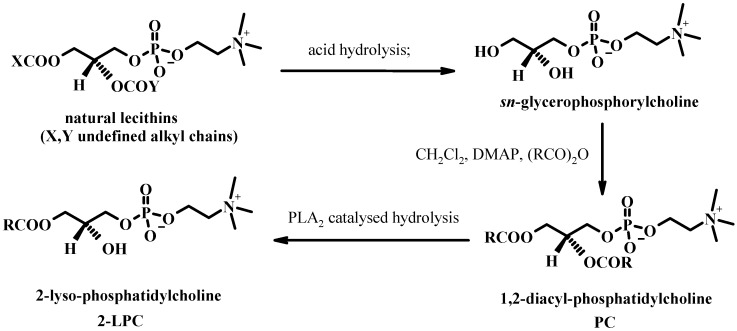
Chemo-enzymatic transformations of natural lecithins into 2-lysophospholipids.

In a consecutive two-step chemo-enzymatic transformation, the 2-lysophosphatidylcholine is obtained from GPC, a fatty acid derivative and an appropriate enzyme in microaqueous system. PLA_2_ can also be used in a chemo-enzymatic process starting from GPC, obtained by hydrolysis of natural lecithins. GPC is acylated with fatty acid anhydride and DMAP and then the obtained PC with the desired acyl chains can be hydrolysed with PLA_2 _to give the desired 2-LPC (see Figure 14).

### 3.2. Use of phospholipase D

Phospholipase D (PLD, phosphatidylcholine phosphatidohydrolase, EC 3.1.4.4) has a very broad distribution in living organisms. It was first isolated in various kind of cabbage and has since been recognized in a number of plants including *ricinus*, castor beans, spinach leaves, soy beans and others [85,86,87,88,89]. Numerous bacterial sources are rich in PLD. The enzyme can be obtained in the culture broth of various *Streptomyces* [90,91,92,93,94,95,96,97]. PLD hydrolyses natural PC to phosphatidic acid PA and choline. PA is an important second messenger in mammalian signal transduction pathways [98]. Many PLDs, in the presence of an alcohol, are able to catalyze the exchange of the polar head group in addition to the hydrolysis product: transphosphatidylation occurs with different degrees of selectivity depending on the enzymatic source, the nature of the alcohol and its concentration. Products of the reaction will be phosphatidic acid (PA) and a new phospholipid (PX) modified in the polar head. Because of this high capacity of transferring the phosphatidyl moiety to an alcohol acceptor in the presence of more than stoichiometric amounts of water in the medium, PLD has then an outstanding position in biocatalytic transformations of PLs [99,100]. The modification of the polar heads of natural or synthetic PLs by PLD is perhaps the most commonly employed enzymatic modification in the preparation of modified PLs. The commercial interests of PLD probably lie in two aspects, namely the enrichment of particular PL species by transphosphatidylation starting from natural lecithin and synthesis of PL derivatives with improved properties or novel compounds for pharmaceutical applications. The transesterification capacity of this enzyme is dependent on the source of PLD and is still under investigation. The increasing number of elucidated PLD sequences as well as the first resolved crystal structure strongly promote the progress in understanding and exploiting the biocatalytic potential of PLD [101,102]. 

In the synthesis of LPLs PLD can be used on a diacylphosphatidylcholine in order to get the PL with the desired polar head group or the PA. Afterwards the PL is submitted to the action of the right phospholipase A_1 _or A_2_ or a lipase in order to get the desired 1-LPL or 2-LPC (see Figure 13). With a similar procedure and starting from commercially available GPC and PLD, fluorogenic analogues of lysophosphatidylcholine were synthesized and evaluated as substrates for enzymatic assays [103]. 

**Figure 15:**
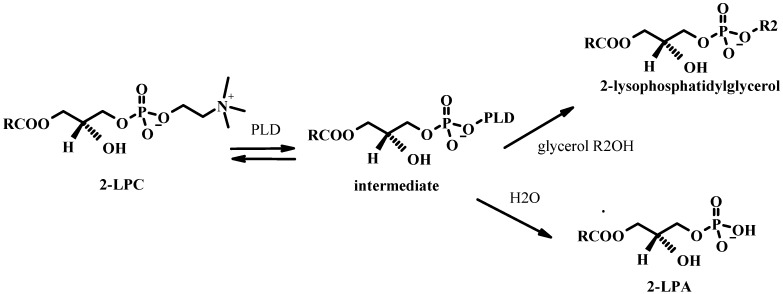
Mechanism for the PLD-catalyzed hydrolysis and transphosphatidylation of 2-LPC with PLD.

LPLs are also the substrates for PLD (see Figure 15). In the absence of an alcohol a crude preparation of cabbage PLD catalyzes the hydrolysis of LPC to LPA. This can happen in one of two ways: i) direct hydrolysis in analogy with the formation of PA from PC; ii) formation of the intermediate cyclic LPA via an intramolecular phosphodiester bond and successive ring opening to [104,105]. In the presence of glycerol, 2-lysophosphatidyl glycerol is formed. The PLD-catalyzed transphosphatidylation reaction of LPC is however about 20 times slower than on PC in a micellar system.

## 4. Concluding Remarks

The regiosecific synthesis of enantiomerically pure LPLs is a challenging task requiring a chiral synthon as starting material and an extensive use of protective group and activating agents. The use of GPC derived from natural PLs is the best strategy if products with the natural absolute configuration are desired. Enzymatic catalysis using a number of phospholipases of different specificity greatly improves the efficiency of the method.
